# Diabetes mellitus aggravates myocardial inflammation and oxidative stress in aortic stenosis: a mechanistic link to HFpEF features

**DOI:** 10.1186/s12933-025-02748-y

**Published:** 2025-05-13

**Authors:** Melissa Herwig, Marcel Sieme, Andrea Kovács, Muchtiar Khan, Andreas Mügge, Wolfgang E. Schmidt, Ferhat Elci, Shan Sasidharan, Peter Haldenwang, Jan Wintrich, Benjamin Sasko, Ibrahim Akin, Máthé Domokos, Francesco Paneni, Ibrahim El-Battrawy, Zoltán V. Varga, Francisca Saraiva, Adelino F. Leite-Moreira, Péter Ferdinandy, Loek van Heerebeek, Inês Falcão-Pires, Nazha Hamdani

**Affiliations:** 1 Medical Faculty, Department of Cellular and Translational Physiology, Institute of Physiology, Molecular and Experimental Cardiology, Institut für Forschung und Lehre (IFL), Ruhr University Bochum, Bochum, Germany; 2 HCEMM-SU Cardiometabolic Immunology Research Group, Department of Pharmacology and Pharmacotherapy, Semmelweis University, Budapest, Hungary; 3 Center for Pharmacology and Drug Research & Development, Semmelweis University, Budapest, Hungary; 4 Department of Cardiology, OLVG, Amsterdam, The Netherlands; 5 Department of Medicine I, St. Josef Hospital, UK RUB, Ruhr University Bochum, Bochum, Germany; 6 HCEMM-SU Cardiovascular Comorbidities Research Group, Department of Pharmacology and Pharmacotherapy, Semmelweis University, Budapest, Hungary; 7 Department of Cardiothoracic Surgery, University Hospital Bergmannsheil Bochum, Bochum, Germany; 8 Medical Department II, Marien Hospital Herne, Ruhr University Bochum, Bochum, Germany; 9 Department of Cardiology, Angiology, Haemostaseology and Medical Intensive Care, University Medical Center Mannheim, Medical Faculty Mannheim, Heidelberg University, Mannheim, Germany; 10 Department of Biophysics and Radiation Biology, Semmelweis University, Budapest, Hungary; 11 In Vivo Imaging Advanced Core Facility, Hungarian Centre of Excellence for Molecular Medicine, Budapest, Hungary; 12 Department of Cardiology, Center for Translational and Experimental Cardiology (CTEC), University Hospital Zurich and University of Zurich, Zurich, Switzerland; 13 University Heart Center, University Hospital Zurich, Zurich, Switzerland; 14 Department of Cardiology, St. Josef-Hospital, UK RUB, Ruhr University Bochum, Bochum, Germany; 15 Department of Surgery and Physiology, Cardiovascular R&D Centre-UnIC@RISE, Faculty of Medicine of the University of Porto, Porto, Portugal; 16 Department of Pharmacology and Pharmacotherapy, Semmelweis University, Budapest, Hungary; 17 Pharmahungary Group, Szeged, Hungary; 18 Department of Physiology, Cardiovascular Research Institute, Maastricht, The Netherlands

**Keywords:** Aortic stenosis, Diabetes mellitus, Cardiomyocyte F_passive_, Heart failure with preserved ejection fraction, Inflammation, Oxidative stress, Protein kinase G, Titin

## Abstract

**Background:**

Patients diagnosed with both aortic stenosis (AS) and diabetes mellitus (DM) encounter a distinctive set of challenges due to the interplay between these two conditions. This study aimed to investigate the effects of DM on the left ventricle in AS patients, specifically focusing on the inflammatory response, oxidative stress, and their implications for cardiomyocyte function, titin phosphorylation, and the nitric oxide (NO)-soluble guanylyl cyclase (sGC)-cyclic guanosine monophosphate (cGMP)-protein kinase G (PKG) signaling pathway.

**Methods and results:**

Left ventricular myocardial biopsies (in total: *n* = 28) were obtained from patients with diabetic AS (*n* = 11) and compared with those from non-diabetic AS patients (*n* = 17). Enzyme-linked immunosorbent assay (ELISA) demonstrated significantly elevated levels of pro-inflammatory mediators, including high mobility group box protein 1 (HMGB1) and calprotectin, as well as receptors associated with the inflammatory response, such as Toll-like receptor 2 (TLR2), 4 (TLR4), and receptor for advanced glycation endproducts (RAGE). These were correlated with an enhanced NOD-like receptor protein 3 (NLRP3) inflammasome and the release of interleukins (IL) 1, 6, and 18 in diabetic AS patients compared to their non-diabetic counterparts. Additionally, in the diabetic AS cohort, there was an increase in oxidative stress markers (hydrogen peroxide (H_2_O_2_), 3-nitrotyrosine, lipid peroxidation (LPO), oxidative glutathione (GSSG)/reduced glutathione (GSH) ratio) within the myocardium and mitochondria, accompanied by impaired NO-sGC-cGMP-PKG signaling, decreased titin phosphorylation, and increased passive stiffness (F_passive_) of cardiomyocytes relative to non-diabetic AS patients. In vitro anti-inflammatory treatment with an IL-6 inhibitor and antioxidant treatment with GSH effectively normalized the elevated F_passive_ observed in AS patients with DM to levels comparable to the non-diabetic group. Furthermore, treatment with PKG and the sodium-glucose cotransporter 2 (SGLT2) inhibitor empagliflozin also resulted in a reduction of F_passive_ in cardiomyocytes from diabetic AS patients, although not to the levels observed in non-diabetic AS patients.

**Conclusion:**

DM exacerbates inflammation and oxidative stress in AS patients, leading to impaired NO-sGC-cGMP-PKG signaling and increased cardiomyocyte F_passive_. These conditions are reminiscent of the pathophysiology of heart failure with preserved ejection fraction (HFpEF). These alterations can be ameliorated through anti-inflammatory and antioxidant therapies, indicating potential therapeutic strategies for diabetic patients suffering from AS.

**Graphical abstract:**

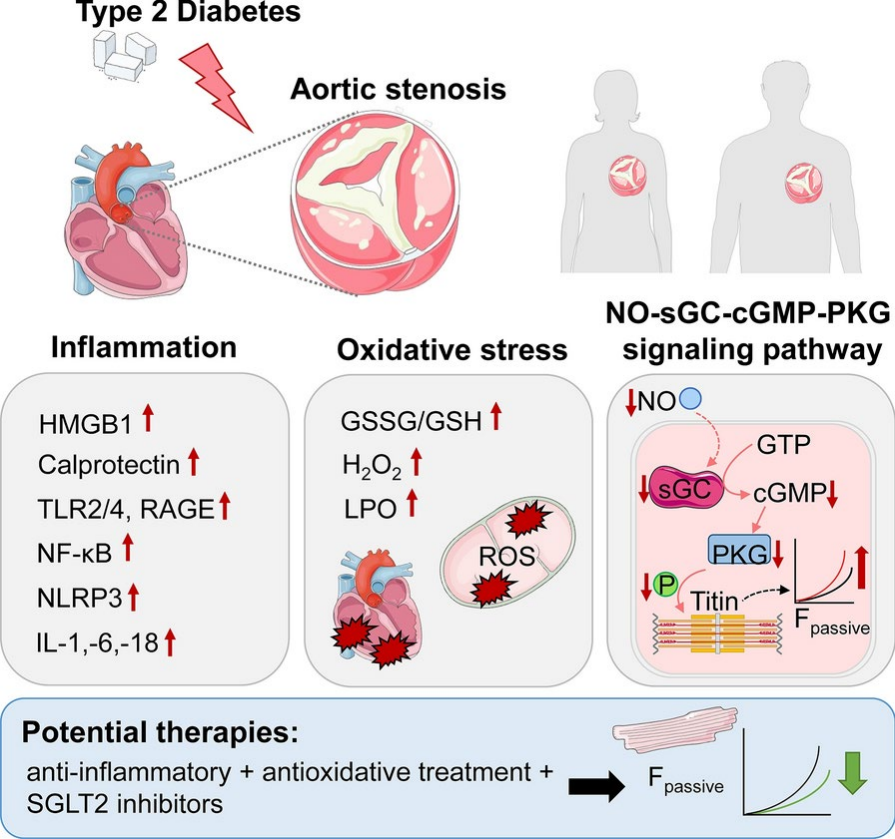

## Introduction

Diabetes mellitus (DM) is well-known as a significant contributor to adverse outcome in patients with aortic stenosis (AS) [1]. Data from the Swedish National Diabetes Registry observed in patients with concomitant AS and DM a faster progression towards death (both all-cause and cardiovascular), heart failure (HF) hospitalization or the need for aortic valve repair (AVR) compared with non-diabetic AS patients [2]. This interplay between DM and AS is probably based on several mechanisms, including an accelerated calcification process within the aortic valve tissue through in-loco inflammation [3, 4] and/or oxidative stress [5, 6], impairment of myocardial perfusion and oxygenation [7], and additive effects on hypertrophic remodelling and myocardial dysfunction beyond known factors of chronic pressure overload [8, 9].

It has been shown previously, that diabetic HF patients had a higher diastolic left ventricular (LV) stiffness irrespective of LV systolic function, which was associated with myocardial deposition of collagen (interstitial fibrosis) and advanced glycation end products (AGEs) as compared to non-diabetic HF patients [10]. In addition, the cardiomyocyte passive stiffness (F_passive_) is increased secondary to an impaired cyclic guanosine monophosphate (cGMP)-protein kinase G (PKG) signaling and elevated protein kinase Cα (PKCα) activity with subsequent changes in titin isoform switch (hypophosphorylation at Ser4099 and hyperphosphorylation at Ser11878) [11]. In the context of AS, concomitant DM further exacerbates the myocardial collagen volume fraction, the deposition of AGEs in arterioles, venules, and capillaries, and the LV end-diastolic stiffness as compared to myocardial biopsies obtained from non-diabetic AS patients [12].

Despite advancements in glucose-lowering therapies, DM- and AS-related myocardial stiffness continue to pose significant therapeutic challenges, underscoring the need for targeted interventions that address both metabolic and mechanical stressors. A comprehensive understanding of the complex interaction between DM and AS is essential for optimizing therapeutic strategies aimed at preserving LV function and preventing the development of HF in this high-risk population.

Increased stiffness of cardiac cells secondary to a reduced soluble guanylyl cyclase (sGC)-cGMP-PKG signaling pathway has been reported in various HF animal models and is also found in human LV myocardial biopsies from patients suffering from HFpEF [13–19]. Reduced cGMP levels and PKG activity with subsequent changes in the phosphorylation of titin´s spring elements exacerbate titin-based passive tension, and consequently elevate cardiomyocyte F_passive_ [14, 15, 20]. As shown previously, this reduced sGC-cGMP-PKG signaling pathway in HFpEF is associated with increased oxidative stress and a pro-inflammatory milieu, whereby several lines of evidence suggest a causal relationship of these changes in the pathophysiology of HFpEF [15, 19, 21–23]. At physiological levels, reactive oxygen species (ROS) play a crucial role in regulating critical cellular functions, including proliferation, differentiation, senescence, migration, apoptosis, and autophagy [18, 22–26]. Additionally, ROS influence key metabolic pathways such as glycolysis, oxidative phosphorylation, and lipid metabolism. These effects are primarily mediated through oxidative modifications of cysteine thiolate groups by hydrogen peroxide (H_2_O_2_) and iron-sulfur clusters by superoxide, resulting in alterations in protein function, localization, and interactions. The pathological condition is characterized by an increase in the activity and expression of ROS-generating enzymes, coupled with a reduction in antioxidant defenses, thereby contributing to disease progression [17, 24–28].

Due to the possible parallels in the development of cardiomyocyte F_passive_ in patients with AS and HFpEF, and the possibility that these cellular mechanisms could be significantly enhanced under the influence of DM, we thought to study in more detail oxidative stress and inflammation pathways in the LV myocardium of AS patients in the presence or absence of DM and to investigate how they contribute to increased cardiomyocyte F_passive_.

## Methods

### Human studies

Twenty-eight AS patients were selected based on retrospective clinical data and the myocardial samples collected during surgical aortic valve replacement (AVR). Informed consent was obtained from all patients. This study was approved by the Ethics Committee of ULS São João Hospital (Ref 109/2022) following the Declaration of Helsinki. Only patients undergoing AVR with severe AS (according to ESC guidelines [29]) and with no more than one stenotic coronary vessel (stenosis > 50%) were considered. Patients with dilated or hypertrophic cardiomyopathies, severe aortic insufficiency and severe cases of mitral stenosis, mitral insufficiency or tricuspid insufficiency were excluded. The diagnosis of DM was considered if the patient was using a glucose-lowering medication and/or insulin or had a fasting plasma glucose 7.0 mmol/L [30]. Clinical evaluation of aortic valve stenosis severity and myocardial structure and function was performed using transthoracic Doppler echocardiography. We derived LV end-systolic volume, LV end-diastolic volume, LV posterior wall thickness, and interventricular septal thickness from 2-dimensional echocardiograms and calculated LV mass index (LVMi) using the Devereux formula following the recent recommendations for cardiac chamber quantification [31]. Peak aortic valve velocity, mean aortic transvalvular pressure gradient and aortic valve area index were derived from Doppler echocardiographic examination of the aortic valve. Mean aortic transvalvular pressure gradient was obtained with the modified Bernoulli equation and aortic valve area index with the standard continuity equation. Perioperative LV myocardial biopsies were procured during AVR. In all patients, LV biopsy material consisted of endomyocardial tissue resected from the LV outflow tract (Morrow procedure) because of concomitant LV outflow tract narrowing. Each myocardial biopsy weighed approximately 1,5–2,5 mg. In total, we used 28 biopsies for different experiments throughout the manuscript. Due to limited biopsy material from each patient, not all experiments could be performed on all samples; hence, the number of patients included in each assay varied based on tissue availability and technical feasibility. Depending on tissue availability, 1–1.5 mg was typically utilized for all the different biochemical assays, including ELISA, nitric oxide quantification, sGC/cGMP/PKG activity assays, CaMKII and PKA activities, and Western blotting. For force measurements, tissue was directly processed for cardiomyocyte isolation.

### Quantification of tissue oxidative stress, inflammatory response, inflammasome and inflammation

Myocardial levels (*n* = 10 samples/group) of inflammatory and oxidative stress markers were measured with enzyme-linked immunosorbent assay (ELISA) and colorimetric assay kits according to manufacturer's instructions. For all ELISA and glutathione assay analyses, myocardial tissues (around 100 ng tissue per sample/assay) were homogenized in a standardized volume of 1xPBS (137 mM NaCl, 2.7 mM KCl, 10 mM Na_2_HPO_4_, 1.8 mM KH_2_PO_4_; all from Sigma-Aldrich) or the assay-specific lysis buffer recommended by the manufacturer. Tissue homogenates were centrifuged for 5 min at 5000 x*g* at 2–8 °C. The supernate was removed and assayed immediately. To reduce background effects, standard curves were created using the same buffer that was used for sample homogenization. Control experiments have been included, demonstrating that the average absorbance of blanks (with and without lysis buffer) remained below 0.05 across all ELISA assays. This confirms negligible background interference. Protein concentration was quantified using the Pierce™ 660 nm assay (Thermo Fisher Scientific, Waltham, MA, USA), and equal protein amounts (typically 20–25 μg per sample) were used per well for each respective assay, in accordance with manufacturer recommendations. The following kits were used in this study: high mobility group box protein 1 (HMGB1) ELISA kit (MBS701378; MyBioSource), calprotectin ELISA kit (EKU02892; Biocompare), Toll-like receptor 2 (TLR2) ELISA kit (RAB0744-1KT; Sigma-Aldrich), TLR4 ELISA kit (RAB1088-1KT; Sigma-Aldrich), receptor for advanced glycation end products (RAGE) ELISA Kit (ab190807; Abcam), nuclear factor kappa B (NF-κB) ELISA Kit (MBS450580; MyBioSource), NOD-like receptor protein 3 (NLRP3) ELISA Kit (ab274401; Abcam), interleukin-(IL) 1 ELISA kit (ab46052; Abcam), IL-6 ELISA kit (ab100772; Abcam) and IL-18 ELISA kit (ab215539; Abcam), 3-nitrotyrosine ELISA kit (ab116691; Abcam), lipid peroxidation (malondialdehyde; LPO) ELISA Kit (ab118970; Abcam).

Total glutathione in myocardial homogenates (around 100 ng tissue per sample) was determined with a colorimetric glutathione assay kit (CS0260, Sigma Aldrich) to assess antioxidant levels. Stable hydrogen peroxide (H_2_O_2_) accumulation was also assessed in myocardial homogenates and mitochondrial fractions using a colorimetric assay (Sigma Aldrich). The GSSG/GSH ratios, LPO and H_2_O_2_ levels were determined both in myocardial homogenates and in the mitochondrial fraction. For fractionation, a subcellular protein fractionation kit (78840; Thermo Fisher Scientific) was used according to manufacturer's instructions. Western blotting for cyclooxygenase-2 (COX) II, COX IV, ATP50, and ATP5a were performed as quality control for mitochondrial enrichment (data not shown).

### 4-HNE Immunohistochemistry

For immunohistochemistry, 6 representative samples per group were used, based on the best-preserved tissue available. After routine formalin-fixed paraffin-embedded (FFPE) specimen processing, 5 μm thick tissue sections were prepared. For immunohistochemistry, deparaffinized sections underwent antigen retrieval (pH = 6 citrate buffer, at 95 °C for 15 min). After blocking endogenous peroxidase activity (3% H_2_O_2_ solution in PBS), the sections were blocked in specific sera (M.O.M. Mouse Ig Blocking Reagent). The primary antibody—mouse anti 4-HNE (HNE-J 2 clone, Jaica, Nikken SEIL Co., Ltd., Japan)—was incubated with the sections overnight in diluted blocking solution at 4 °C. After primary antibody incubations, the sections were washed three times in 1xPBS and incubated for 1 h with an M.O.M. ImmPRESS anti-mouse Ig reagent conjugated with a peroxidase polymer (Vector Laboratories, Burlingame, CA, USA). Secondary antibodies were washed 3 times for 10 min and the specific signal was developed with diaminobenzidine (ImmPACT DAB EqV Peroxidase (HRP) Substrate, Vector Laboratories, Burlingame, CA, USA). All the stainings were visualized and images were captured with a 6.3 × objective on a Leica LMD6 microscope (Wetzlar, Germany).

### Force measurements

Force measurements were performed on single de-membranated cardiomyocytes (*n* = 30–36/5 cardiomyocytes/hearts per group) as described before [14, 32]. In brief, LV samples (approximately 0.3 mg wet weight) were de-frozen in relaxing solution (containing in mM: 1.0 free Mg^2+^; 100 KCl; 2.0 EGTA; 4.0 Mg-ATP; 10 imidazole; pH 7.0), mechanically disrupted and incubated for 5 min in relaxing solution supplemented with 0.5% Triton X-100 (all from Sigma-Aldrich). The cell suspension was washed 5 times in relaxing solution. Single cardiomyocytes were selected under an inverted microscope (Zeiss Axiovert 135, 40 × objective; Carl Zeiss AG Corp, Oberkochen, Germany) and attached with silicone adhesive between a force transducer and a high-speed length controller (piezoelectric motor) as part of a “Permeabilized Myocyte Test System” (1600A; with force transducer 403A; Aurora Scientific, Aurora, Ontario, Canada). Cardiomyocyte Ca^2+^-independent passive force (F_passive_) was measured in relaxing buffer at room temperature (RT) within a sarcomere length (SL) range between 1.8 and 2.4 μm. Force values were normalized to myocyte cross-sectional area calculated from the diameter of the cells, assuming a circular shape. Intact cardiomyocytes were incubated for 30 to 40 min in relaxing solution supplemented with either (1) an inhibitor of interleukin (IL-) 6 (Siltuximab, 0.015 mL/L, EUSA Pharma Ltd, Hemel Hempstead, Hertfordschire, UK) (2) the antioxidant reduced glutathione (GSH) 30 min (10 mM; Sigma-Aldrich Merck KGaA, St. Louis, MO, USA), (3) the mitochondria-targeted superoxide dismutase mimetic MitoTEMPO (10 µM; Sigma-Aldrich Merck KGaA, St. Louis, MO, USA), (4) PKG1α (batch 034K1336, 0.1 U/mL; Sigma-Aldrich), cGMP (10 µM; Sigma-Aldrich Merck KGaA, St. Louis, MO, USA) and DTT (6 mM; Sigma-Aldrich Merck KGaA, St. Louis, MO, USA), (5) empagliflozin (EMPA) (0.5 µmol/L; Sigma-Aldrich Merck KGaA, St. Louis, MO, USA), (6) protein kinase A (PKA, 100 U/mL; batch-12K7495; Sigma-Aldrich Merck KGaA, St. Louis, MO, USA), or (7) CaMKII (CaMKIIδ; 0.6 μg/mL in the calmodulin-containing buffer; Merck Millipore, Burlington, MA, USA). Cardiomyocyte F_passive_ was thereafter measured in single skinned cells within a SL range between 1.8 and 2.4 μm as described above.

### Titin-analysis by Western blot

Sodium Dodecyl Sulfate (SDS)-Polyacrylamide gel electrophoresis (PAGE) was performed to separate titin as previously described [14, 20]. LV tissue samples (*n* = 10 samples/group; approximately 100 ng of tissue per sample) were homogenized in a modified Laemmli buffer (0.05 M Tris–HCl pH 6.8, 8 M urea, 2 M thiourea, 3% SDS (*w*/*v*), 0.03% ServaBlue (*w*/*v*), 10% (*v*/*v*) glycerol, 75 mM DTT). After 20 min incubation on ice, samples were heated for 3 min at 96 °C and centrifuged for 3 min at 14,000 rpm. The concentration of the samples was determined using the Pierce™ 660 nm protein assay (Thermo Fisher Scientific, Waltham, MA, USA). Samples (20 µg) were separated via agarose strengthened 2% SDS-PAGE. Gels were run at 2–4 mA constant current per gel for 16 h. After SDS-PAGE, the gels were blotted onto polyvinylidene difluoride (PVDF) membranes (Immobilon-P 0.45 μm; Merck Millipore, Burlington, MA, USA). Blots were blocked with 5% bovine serum albumin (BSA) in 1xTris-buffered saline with Tween (1xTBST; 50 mM Tris pH 8.0, 150 mM NaCl, 0.05% Tween-20; all from Sigma-Aldrich) for 1 h at RT and subsequently incubated with either anti-phospho serine (Ser)/threonine (Thr) antibody (dilution 1:500; ECM Biosciences LLC, Versailles, KY, United States) to assess total titin phosphorylation or the following primary phosphosite-specific anti-titin antibodies (custom-made by Eurogentec (Seraing, Belgium [32]) overnight at 4 °C:

- Anti-phospho-N2Bus-titin (Ser4010) against EEGKS(PO3H2)LSFPLA (dilution 1:500);
- Anti-phospho-N2Bus-titin (Ser4062) against DLLS(PO 3H2)KESLLS (dilution 1:100);
- Anti-phospho-N2Bus-titin (Ser4099) against LFS(PO3H2)EWLRNI (dilution 1:500);

After washing with 1xTBST, primary antibodies were detected with horse radish peroxidase (HRP)-conjugated secondary goat anti-rabbit antibody (dilution 1:10,000; OriGene Technologies GmbH) and enhanced chemiluminescence (Clarity Western ECL Substrate, BioRad, Munich, Germany). Imaging was carried out with a ChemiDoc Imaging system (BioRad). Chemiluminescence signals were normalized to signals obtained from Coomassie-stained PVDF membranes referring to the entire protein amount transferred. Stained protein bands were quantified via densitometry using the Multi Gauge V3.2 software (FUJIFILM Corp, Minato, Tokyo, Japan).

### Small protein analysis by Western blot

SDS-PAGE was performed to separate small proteins as previously described [15, 33]. LV tissue samples (*n* = 8 samples/group; approximately 100 ng of tissue per sample) were homogenized in a modified Laemmli buffer (see above). After 20 min incubation on ice, samples were heated for 3 min at 96 °C and centrifuged for 3 min at 14,000 rpm. The concentration of the samples was determined using the Pierce™ 660 nm protein assay (Thermo Fisher Scientific, Waltham, MA, USA). Samples (20 µg) were separated 10% SDS-PAGE. Gels were run at 90 V for 20 min followed by 125 V for about 90 min. After SDS-PAGE, the gels were blotted onto PVDF membranes (Immobilon-P 0.45 μm; Merck Millipore, Burlington, MA, USA). Blots were blocked with 5% BSA in 1xTBST (50 mM Tris pH 8.0, 150 mM NaCl, 0.05% Tween-20; all from Sigma-Aldrich) for 1 h at RT and subsequently incubated with anti-CaMKIIδ antibody (#PA5-22168; Invitrogen, Life Technologies GmbH, Darmstadt, Germany) overnight at 4 °C. After washing with 1xTBST, primary antibodies were detected with HRP-conjugated secondary goat anti-rabbit antibody (dilution 1:10,000; OriGene Technologies GmbH) and enhanced chemiluminescence (Clarity Western ECL Substrate, BioRad, Munich, Germany). Imaging was carried out with a ChemiDoc Imaging system (BioRad). Finally, the signals obtained for the amounts of total protein were normalized to signals obtained from anti-β-actin (#A1979, Sigma-Aldrich Merck KGaA, St. Louis, MO, US) stained membranes. Stained protein bands were quantified via densitometry using the Multi Gauge V3.2 software (FUJIFILM Corp, Minato, Tokyo, Japan).

### Quantification of nitric oxide (NO) level

The levels of NO were measured by means of a colorimetric assay kit (BioVision Inc, Milpitas, CA, USA) providing the measurement of total nitrate/nitrite as previously described [15, 19]. NO production was measured in tissue homogenates. Briefly, LV tissue samples (*n* = 10 LV samples/group; approximately 100 ng of tissue per sample) were initially treated with trichloroacetic acid (8 g in 80 mL acetone; Sigma-Aldrich) and washed with 1 mL 0.2% dithiothreitol (DTT) for protein precipitation and the removal of interfering proteins. Tissue samples were homogenized in 1% SDS sample buffer (Tri-distilled water: 8.47 mL; glycerol: 2.1 mL; 10% SDS: 1.4 mL; 0.5 M Tris–HCl (pH 6.8): 1.75 mL; brome-phenol blue: 0.28 mL; DTT: 32.4 mg; all from Sigma-Aldrich). After homogenization, tissue samples underwent sonication and were subsequently centrifuged at 14,000 *g* for 15 min at 2–8 °C. Supernatants (free of SDS and brome-phenol blue) containing equal amounts of total protein (typically 20–25 μg per sample were used per well) were analysed for NO concentration. The assay buffer provided with the NO detection kit was used to resuspend the final samples and conduct the measurements. In the first step, nitrate was converted to nitrite using a nitrase reductase. The second step involves the conversion of nitrite into an azochromophore by the Griess reagents, which reflects the NO content in the tissue samples. Nitrite levels could be measured independently from nitrate by omitting the first step. The absorbance of samples was measured at 540 nm using a plate reader. An assay buffer was used to generate a standard curve from which the absorbances of the samples could be translated into the nitrite and nitrate concentrations.

### Measurement of soluble guanylyl cyclase (sGC) activity

The activity of sGC of was assessed by means of a colorimetric assay kit (MyBioSource) as previously described [15]. In brief, approximately 100 ng of tissue (*n* = 10 LV samples/group) were rinsed and homogenised in 1xPBS and stored overnight in − 20 °C. These tissue samples underwent sonication and were subsequently centrifuged at 14,000 *g* for 15 min at 2–8 °C. Supernatants containing equal amounts of total protein (typically 20–25 μg per sample were used per well) were analysed for sGC activity according to manufacturer's instructions. The absorbance of samples was measured at 570 nm using a plate reader.

### Measurement of myocardial cyclic guanosine monophosphate (cGMP) level

Myocardial cGMP level were measured according to previous protocols [13, 21]. Briefly, cGMP was determined in LV homogenates (*n* = 8 samples/group; approximately 100 ng of tissue per sample) by means of a parameter cGMP assay immunoassay kit (R&D Systems, Minneapolis, MN, United States), in which cGMP present in the homogenate competes with a fixed amount of HRP-labeled cGMP for sites on a rabbit polyclonal antibody. These homogenates were diluted in cell lysis buffer, and 100 µL of 0.025 µg/µL protein aliquots were measured according to manufacturer's instructions. Results of duplicate determinations were averaged and expressed as μg/μL.

### Measurement of myocardial protein kinase G (PKG) activity

LV tissues samples (*n* = 8 samples/group; approximately 100 ng of tissue per sample) were homogenized in 25 mM Tris–HCl (pH 7.4), 1 mM EDTA, 2 mM EGTA, 5 mM DTT, 0.05% Triton X-100 and protease inhibitor cocktail (all from Sigma-Aldrich) and centrifuged for 5 min. Supernatants containing equal amounts of total protein were analysed for PKG activity (typically 20–25 μg per sample were used per well) as described previously [21]. In brief, reaction mixtures were incubated at 30 °C for 10 min. Reaction mixtures contained 40 mM Tris–HCl (pH 7.4), 20 mM Mg(CH_3_COO)_2_, 0.2 mM [^32^P] adenosine triphosphate (ATP) (500–1,000 cpm pM–1; Amersham PLC, Little Chalfont, UK), 113 mg/mL heptapeptide (RKRSRAE) and 3 μM cGMP (both from Promega Corp, Madison, WI, USA), and a highly specific inhibitor of cyclic adenosine monophosphate-dependent protein kinase (5–24; Calbiochem, San Diego, CA, USA). The reaction was terminated by spotting 70 μL onto Whatman P-81 filters (MACHEREY–NAGEL). Samples were subsequently incubated and washed with 75 mM H_3_PO_4_ for 5 min to remove unbound ATP. Filters were then washed with 100% ethanol and air dried before quantification. PKG activity was quantified using a Wallac 1409 Liquid Scintillation Counter (Hidex Oy, Turku, Finland). Specific activity of PKG was expressed as pM of ^32^P incorporated into the substrate (pM/min/mg protein).

### Measurement of myocardial protein kinase A (PKA) activity

PKA activity (*n* = 8 samples/group; approximately 100 ng of tissue per sample) was analyzed using a non-radioactive PKA kinase activity assay kit (Enzo Life Science) as previously described [15]. Samples were homogenized in cell lysis buffer (20 mmol/L MOPS, 50 mmol/L β-glycerolphosphate, 50 mmol/L sodium fluoride, 1 mmol/L sodium vanadate, 5 mmol/L EGTA, 2 mmol/L EDTA, 1% NP40, 1 mmol/L DTT, 1 mmol/L benzamidine, 1 mmol/L phenylmethanesulphonylfluoride, and 10 μg/mL leupeptin and aprotinin, each). Supernatants were collected after centrifugation at 13,000 rpm for 30 min. Supernatants containing equal amounts of total protein (30 ng/μL protein aliquots were assayed according to manufacturer’s instructions; typically 20–25 μg per sample were used per well) were added into the appropriate wells of the PKA substrates microliter plate. PKA kinase reaction was initiated by addition of ATP, and samples were subsequently incubated at 30 °C for 90 min. Phosphorylated peptide substrates were recognized by phospho-specific substrate antibody. The phospho-specific antibody was subsequently bound by a peroxidase-conjugated secondary antibody anti-rabbit IgG-HRP. The assay was developed with tetramethylbenzidine, and the intensity of the colour was measured in a microplate reader at 450 nm. Results of triplicate determinations were averaged, and specific activity of PKA was expressed as ng/μL.

### Measurement of myocardial calcium-calmodulin dependent kinase II (CaMKII) activity

CaMKII activity (*n* = 8 samples/group; approximately 100 ng of tissue per sample) was determined using a CycLex® CaMKII assay kit (CY-1173; MBL Corporation, MA, United States) according to the manufacturer’s guidelines and previously described [19]. Briefly, frozen LV heart samples were homogenized in sample buffer containing 15% glycerol, 62.5 mmol/L Tris; pH 6.8; 1% (w/v) SDS, protease inhibitor, and protein phosphatase inhibitor, all prepared in distilled H_2_O. Homogenates were centrifuged at 10,000 ×*g* for 15 min at 4 °C. The supernatant was removed and stored at − 80 °C. Protein samples were loaded onto microtiter wells (concentration, ∼ 2.0 μg/well) coated with CaMKII specific substrate, syntide-2, along with kinase reaction buffer with or without Ca^2+^/calmodulin. To quantify CaMKII activity, a standard curve correlating the amount of active CaMKII and the level of phosphorylation of syntide-2 was constructed.

### Statistics

Unpaired two-tailed Student´s t-test was performed to analyze the clinical data. For the ELISA assays, activity assays and Western blot data, we used estimation plots which show on the left axis the data as box and whisker plots (median, 25th to 75th percentiles, minimum and maximum) in order to present individual points in each group. The dashed lines represent the mean values of both groups. The effect size meaning the difference between the means (mean ± SEM) is shown on the right axis. The *P* values are from unpaired Student’s t-test; *P < 0.05, **P < 0.01, ***P < 0.001, ****P < 0.0001. For the analysis of force measurements which involves parametric data comparing more than two groups one-way ANOVA was used. *P*-values were adjusted for multiple comparisons using the Tukey method; significant comparisons include *P < 0.05 AS-DM versus AS+DM, ^‡^*P* < 0.05 AS-DM versus AS-DM after treatment, †*P* < 0.05 AS+DM versus AS+DM after treatment by one-way ANOVA. For analysis of proportions Fisher's exact test was used. Analysis was performed using GraphPad Prism 10. *P* values are two-sided and considered statistically significant if *P* < 0.05. We applied t-tests or ANOVA with Tukey correction under the assumption of normality which was confirmed by Shapiro–Wilk tests for each dataset. Normality testing was performed to justify the use of statistical conclusions based on the mean, despite the median-based presentation in the box and whisker plots.

## Results

### Diabetes mellitus enhances upstream signaling pathways of inflammasome and inflammation, increasing cardiomyocyte passive stiffness

This study aims to investigate the effects of DM on AS by utilizing LV myocardial samples from diabetic patients with AS and comparing them to non-diabetic AS patients. Table 1 compares the clinical and echocardiographic characteristics of AS and AS+DM patients. As evident from the aortic valve area index (AVAi) and mean aortic transvalvular pressure gradient (mean ATPG), the severity of AS was comparable in AS and AS+DM patients. LV hypertrophy was present in both groups, as evidenced by the similar left ventricular mass index (LVMi) between both groups. The groups were comparable regarding risk factors such as age, body mass index (BMI), smoking history, hypertension and medication(Table 1).The ejection fraction (EF) differed significantly between both groups. In addition, no statistical difference between sex was observed between both groups.

**Table 1:** Clinical characteristics and echocardiographic measures in patients with aortic stenosis (AS) and concomitant AS and diabetes mellitus (AS+DM)

Parameter	AS (n = 17)	AS+DM (n = 11)	*P* value
Age (mean ± SEM)	69.9 ± 2.11	76.3 ± 4.0	0.5960
Sex (male)	11 (64.7%)	5 (45.5%)	0.8870
BMI (kg/m^2^) (mean ± SEM)	27.7 ± 1.43	29.4 ± 1.62	0.2595
Hypertension (n)	12 (70.6%)	5 (45.5%)	0.0553
Diabetes mellitus (n)	0 (0%)	11 (100%)	–
Smoking history (n)	1 (7.1%)	1 (9.1%)	0.7750
NYHA classification			0.2354
0	2 (11.8%)	0 (0%)	
1	0 (0%)	0 (0%)	
2	8 (47.1%)	5 (45.5%)	
3	3 (17.6%)	3 (27.3%)	
Not classified	4 (23.5%)	3 (27.3%)	
LVEF	64.6 ± 1.76	59.1 ± 3.00	0.0380
Statins (n)	7 (41.2%)	6 (54.5%)	0.9741
Beta-blockers (n)	6 (35.3%)	3 (27.3%)	0.1459
ACEi (n)	4 (23.5%)	4 (36.4%)	0.9202
AT2Ri (n)	2 (11.8%)	3 (27.3%)	0.4965
Diuretics (n)	9 (52.94%)	6 (54.5%)	0.2658
MRA (n)	3 (17.6%)	3 (27.3%)	1.0000
AVAi, cm^2^/m^2^ (mean ± SEM)	0.45 ± 0.04	0.40 ± 0.02	0.0675
Max ATPG, mmHg (mean ± SEM)	96.93 ± 8.85	80.4 ± 6.85	0.2492
Mean ATPG, mmHg (mean ± SEM)	60.3 ± 5.2	49.6 ± 3.61	0.1187
LVEDD, mm (mean ± SEM)	50.9 ± 1.73	49.1 ± 2.30	0.3895
IVST, mm (mean ± SEM)	14.1 ± 0.57	15.3 ± 1.16	0.3160
PWT, mm (mean ± SEM)	11.7 ± 0.53	12.4 ± 0.65	0.4289
RWT (mean ± SEM)	0.48 ± 0.03	0.46 ± 0.06	0.4367
LVMi, g/m^2^ (mean ± SEM)	146 ± 10.9	156.8 ± 18.3	0.5945

Values are represented as mean ± SEM. *P* values are from unpaired two-tailed Student´s t-test

*ACEi* angiotensin-converting enzyme inhibitors; *ATPG* aortic transvalvular pressure gradient; *AT2Ri* angiotensin II receptor inhibitors; *AVAi* aortic valve area, indexed to body surface area; *BMI* body mass index; *CAD* coronary artery disease; *COPD* chronic obstructive pulmonary disease; *IVST* interventricular septal thickness; *LVEDD* left ventricle end-diastolic dimension; *LVEF* left ventricle ejection fraction; *LVMi* left ventricle mass, indexed to body surface area; *MRA* mineralocorticoid receptor antagonists; *Peak Ao* peak aortic valve velocity; *PWT* posterior wall thickness; *RWT* relative wall thickness

**Fig. 1:**
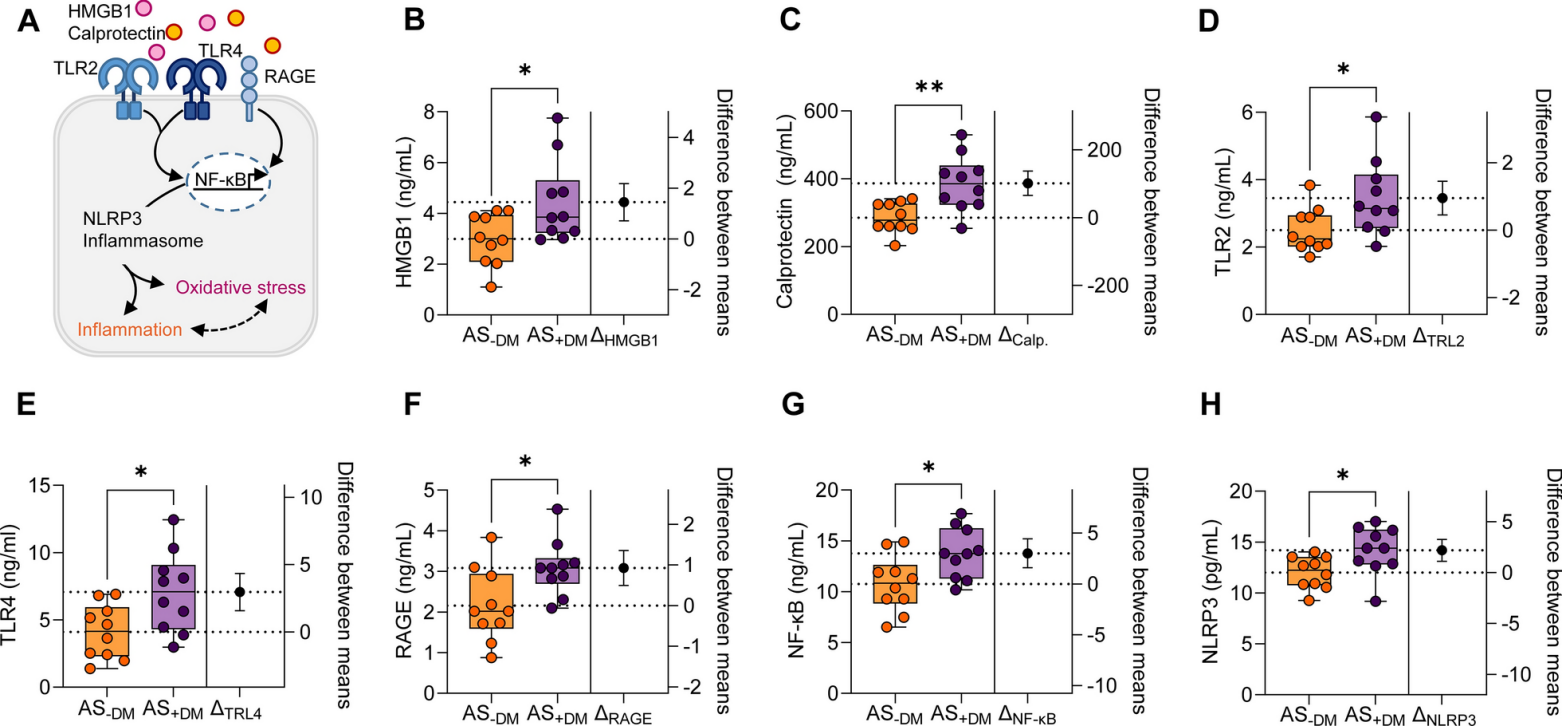
Upstream signaling pathways of inflammation and oxidative stress in left ventricular biopsies from patients with aortic stenosis (AS), with (+DM) and without concomitant diabetes (−DM). **A** Schematic representation of intracellular oxidative and inflammatory signaling pathways. HMGB1: High mobility group box protein 1; NF-κB: nuclear factor kappa B; NLRP3: NOD-like receptor protein 3; RAGE: receptor for advanced glycation end products; TLR: Toll-like receptor. **B** HMGB1 levels. **C** Calprotectin levels. **D** TLR2 levels. **E** TLR4 levels. **F** RAGE levels. **G** NF-κB levels. **H.** NLRP3 levels. Box and whisker plots (median, 25th to 75th percentiles, minimum, and maximum) are utilized on the left axis to represent selected parameters in each group (*n* = 10 samples per group). The dashed lines represent the mean values of both groups. *P*-values are derived from an unpaired t-test; *P < 0.05, **P < 0.01.The right axis shows the difference between the means ± SEM

We examined the primary signaling pathways and receptors involved in the induction of inflammation and oxidative stress (Fig. 1A). The levels of high mobility group box protein 1 (HMGB1; Fig. 1B; AS−DM: mean ± SEM: 2.991 ± 0.3215 ng/mL; AS+DM: 4.439 ± 0.5136 ng/mL) and calprotectin (Fig. 1C; AS−DM: mean ± SEM: 285.6 ± 14.28 ng/mL; AS+DM: 386.8 ± 25.94 ng/mL) were significantly elevated in the LV myocardium of AS patients with DM as compared to their non-diabetic counterparts. These two pro-inflammatory mediators have the capacity to trigger intracellular inflammatory signaling processes through interactions with three distinct receptors: toll-like receptor 2 (TLR2), TLR4, and the receptor for advanced glycation end products (RAGE; Fig. 1A), among others. It is well-documented that the accumulation of AGEs plays a pivotal role in the progression of AS [34]. The levels of TLR2 (Fig. 1D; AS−DM: mean ± SEM: 2.504 ± 0.2063 ng/mL; AS+DM: 3.455 ± 0.3562 ng/mL), TLR4 (Fig. 1E; AS−DM: mean ± SEM: 4.111 ± 0.6366 ng/mL; AS+DM: 7.067 ± 0.9429 ng/mL), and RAGE (Fig. 1F; AS−DM: mean ± SEM: 2.158 ± 0.2820 ng/mL; AS+DM: 3.084 ± 0.2144 ng/mL) were significantly heightened in diabetic AS patients compared to non-diabetic AS patients. Furthermore, downstream components of the cardiac inflammasome, including nuclear factor kappa B (NF-κB; Fig. 1G; AS−DM: mean ± SEM: 10.77 ± 0.8726 ng/mL; AS+DM: 13.77 ± 0.7853 ng/mL) and NOD-like receptor protein 3 (NLRP3; Fig. 1H; AS−DM: mean ± SEM: 12.00 ± 0.4961 pg/mL; AS+DM: 14.19 ± 0.7383 pg/mL), were also significantly increased in diabetic AS compared to non-diabetic AS patients.

**Fig. 2:**
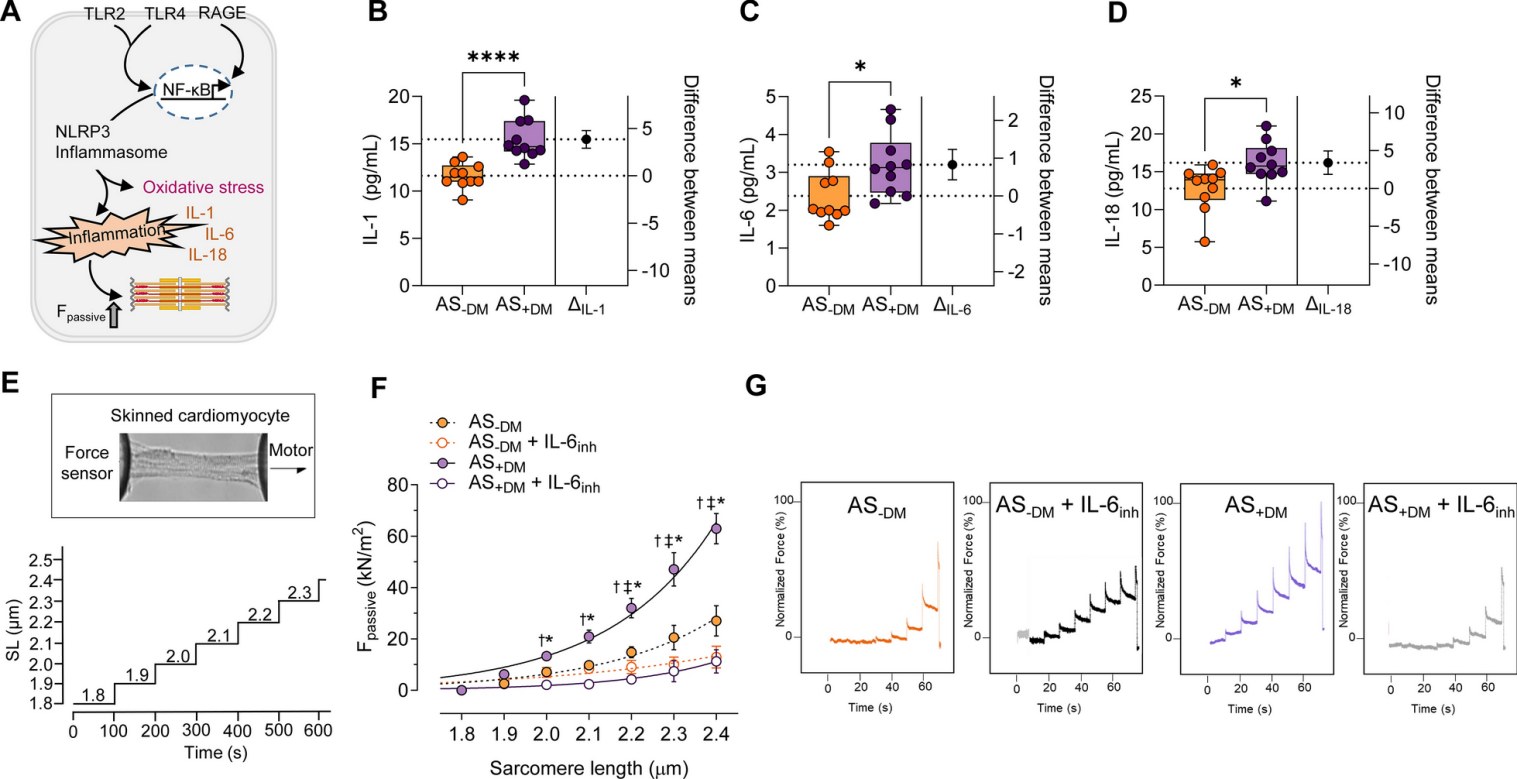
Inflammatory markers and cardiomyocyte passive stiffness in left ventricular biopsies from patients with aortic stenosis (AS), with (+DM) and without concomitant diabetes (−DM). **A** Schematic representation of intracellular inflammatory signaling pathways. F_passive_: passive stiffness; HMGB1: high mobility group box protein 1; IL: interleukin; NF-κB: nuclear factor kappa B; NLRP3: NOD-like receptor protein 3; RAGE: receptor for advanced glycation end products; TLR: Toll-like receptor. **B**–**D** Levels of IL-1, IL-6, and IL-18. Box and whisker plots (median, 25th to 75th percentiles, minimum, and maximum) are displayed on the left axis to represent selected parameters in each group (*n* = 10 samples per group). The dashed lines represent the mean values of both groups. *P*-values are derived from unpaired t-tests; *P < *0.05,* ****P < 0.0001. The right axis shows the difference between the means ± SEM. **E** Stretch protocol; SL: sarcomere length. **F** Cardiomyocyte F_passive_ at SL 1.8–2.4 µm in the presence or absence of in vitro treatment with an inhibitor of IL-6 (IL-6_inh_). Curves are second-order polynomial fits to the means (± SEM; *n* = 30–36/5 cardiomyocytes/heart per group), *P < 0.05 AS−DM versus AS+DM, ^‡^*P* < 0.05 AS−DM alone versus AS−DM after IL-6_inh_, †*P* < 0.05 AS+DM alone versus AS+DM after IL-6_inh_ by one-way ANOVA. *P*-values were corrected for multiple comparisons by the Tukey method. **G.** Original recordings

The NLRP3 inflammasome is critical for the host immune response and the processing of various pro-inflammatory cytokines, including interleukins (IL; Fig. 2A). The augmented NLRP3 inflammasome complex was associated with significantly elevated levels of IL-1 (Fig. 2B; AS−DM: mean ± SEM: 11.61 ± 0.4117 pg/mL; AS+DM: 15.46 ± 0.6483 pg/mL), IL-6 (Fig. 2C; AS−DM: mean ± SEM: 2.375 ± 0.2073 pg/mL; AS+DM: 3.203 ± 0.2590 pg/mL), and IL-18 (Fig. 2D; AS−DM: mean ± SEM: 12.79 ± 0.9408 pg/mL; AS+DM: 16.20 ± 0.8725 pg/mL) in diabetic AS patients compared to non-diabetic AS patients. It is recognized that inflammation is closely linked to oxidative stress and induces cardiac microvascular dysfunction, which subsequently leads to impaired cardiomyocyte function and increased stiffness. We measured the calcium (Ca^2+^)-independent cardiomyocyte passive stiffness (F_passive_) within a sarcomere length (SL) range of 1.8 to 2.4 µm (Fig. 2E). The F_passive_ of diabetic AS patients exhibited a significantly steeper increase at SLs beyond 2.0 µm compared to non-diabetic AS patients (Fig. 2F). In vitro treatment with an inhibitor of IL-6 (IL-6_inh_) significantly reduced the cardiomyocyte F_passive_ at SLs ranging from 2.0 to 2.4 µm in diabetic AS patients, whereas this effect was only significant at SLs beyond 2.2 µm in the non-diabetic group (Fig. 2F). The force recordings for both groups before and after treatment with IL-6_inh_ are illustrated in Fig. 2G.

### Severe myocardial and mitochondrial oxidative stress and lipid peroxidation in aortic stenosis patients with diabetes mellitus

**Fig. 3:**
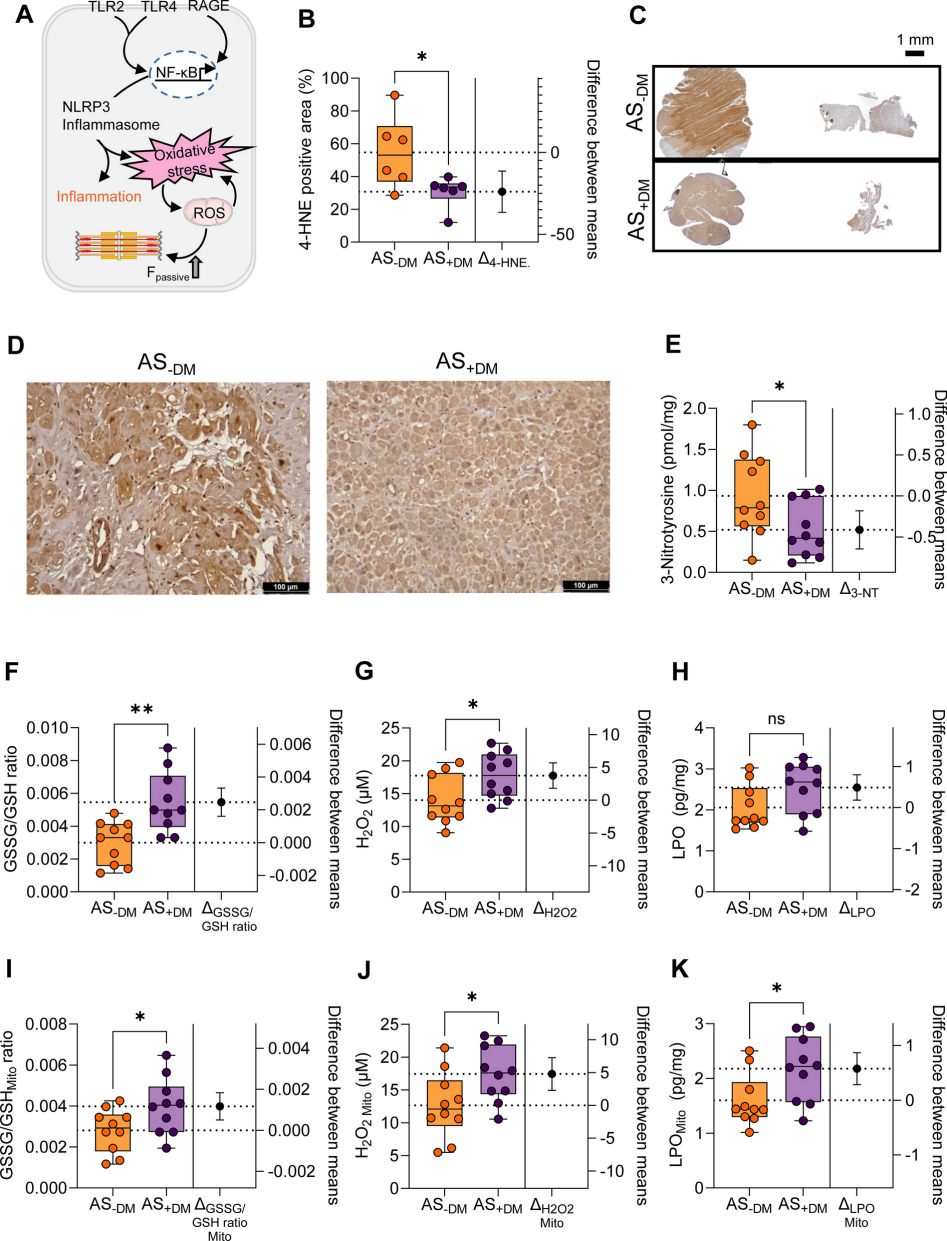
Oxidative stress markers in left ventricular biopsies from patients with aortic stenosis (AS), with (+DM) and without concomitant diabetes (−DM). **A** Schematic representation of intracellular oxidative signaling pathways. F_passive_: passive stiffness; NF-κB: nuclear factor kappa B; NLRP3: NOD-like receptor protein 3; RAGE: receptor for advanced glycation end products; ROS: reactive oxygen species; TLR: Toll-like receptor. **B** Quantification of immunostaining of peroxidation (4-HNE, 4-hydroxynonenal). **C** Whole scan sections of 4-HNE immunostaining (scale bar 1 mm) showing the overall difference in staining. **D** 4-HNE immunostaining at higher magnitude (scale bar 100 µm). **E** Measurement of 3-nitrotyrosine (3-NT) levels. **F** Ratio of myocardial oxidized glutathione (GSSG) over reduced glutathione (GSH). **G** Measurement of myocardial hydrogen peroxide (H_2_O_2_) levels. **H** Measurement of myocardial lipid oxidation (LPO) levels. **I** Ratio of GSSG to GSH in mitochondria (Mito). **J** Mitochondrial levels of H_2_O_2_. **K** Mitochondrial levels of LPO. Box and whisker plots (median, 25th to 75th percentiles, minimum and maximum) are used on the left axis to represent selected parameters across each group (*n* = 10 samples per group). The dashed lines represent the mean values of both groups. P-values are from unpaired t-test; *P < 0.05, **P < 0.01. The right axis shows the difference between the means ± SEM

In this study, we aimed to analyze oxidative stress parameters in the myocardium and mitochondria, given that oxidative stress is strongly associated with inflammation and vice versa. Both factors are known to contribute to the progression of cardiomyocyte dysfunction (Fig. 3A). Staining for 4-hydroxynonenal (4-HNE), a product of lipid oxidation and peroxidation, revealed that lipid peroxidation (LPO) is less prevalent in diabetic patients with AS compared to their non-diabetic counterparts (Fig. 3B-3D; AS−DM: mean ± SEM: 54.75 ± 8.951%; AS+DM: 30.77 ± 3.922%). Figure 3C shows the overall difference in staining intensity for 4-HNE, while Fig. 3D shows 4-HNE staining at a higher resolution.

In the cardiac tissue of diabetic AS patients, we observed a reduction in 3-nitrotyrosine levels compared to the non-diabetic group (Fig. 3E; AS−DM: mean ± SEM: 0.9315 ± 0.1595 pmol/mg; AS+DM: 0.5181 ± 0.1061 pmol/mg). An overall increase was noted in the oxidative stress parameters of the oxidized glutathione (GSSG)/reduced glutathione (GSH) ratio (Fig. 3F; AS−DM: mean ± SEM: 0.002996 ± 0.0004070; AS+DM: 0.005461 ± 0.0005801) and hydrogen peroxide (H_2_O_2_; Fig. 3G; AS−DM: mean ± SEM: 14.00 ± 1.154 µM; AS+DM: 17.75 ± 1.097 µM) in the myocardium of the diabetic AS cohort, whereas the level of LPO (Fig. 3H; AS−DM: mean ± SEM: 2.056 ± 0.1691 pg/mg; AS+DM: 2.546 ± 0.1923 pg/mg) remained unchanged between the two groups.

**Fig. 4:**
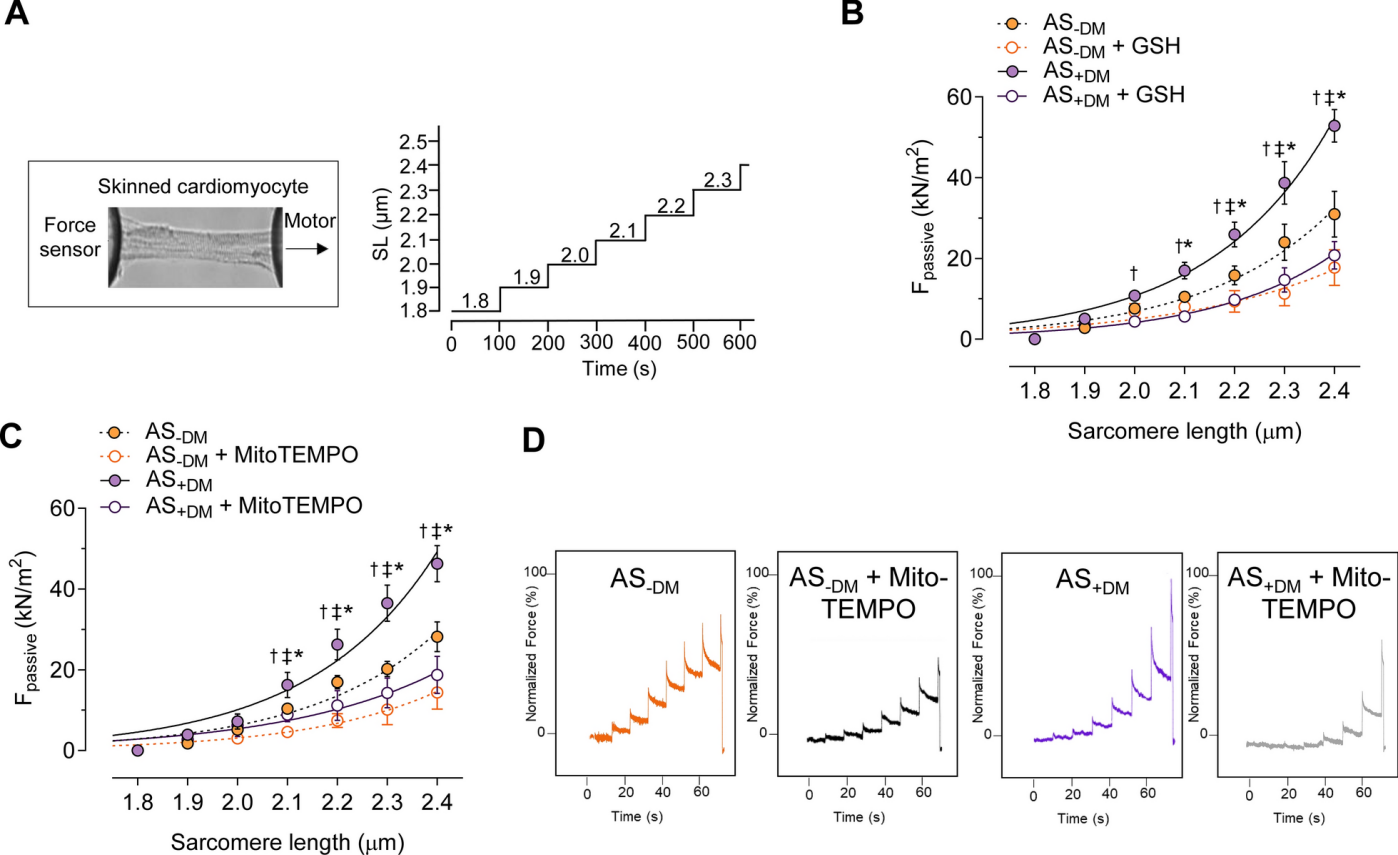
Cardiomyocyte F_passive_ before and after antioxidant treatment of left ventricular biopsies from patients with aortic stenosis (AS), with (+DM) and without concomitant diabetes (−DM). **A** Stretch protocol; SL: sarcomere length. **B** F_passive_ at sarcomere length 1.8–2.4 µm in the presence or absence of reduced glutathione (GSH)._−DM__+DM_ **C** F_passive_ at SL 1.8–2.4 µm in the presence or absence of the mitochondria-targeted superoxide dismutase mimetic MitoTEMPO. Curves are second-order polynomial fits to the means (± SEM; *n* = 30–36/5 cardiomyocytes/heart per group), *P < 0.05 AS−DM versus AS+DM, ^‡^*P* < 0.05 AS−DM alone versus AS−DM alone after GSH or MitoTEMPO treatment, †*P* < 0.05 AS+DM alone versus AS+DM after GSH or MitoTEMPO treatment by one-way ANOVA. *P*-values were corrected for multiple comparisons by the Tukey method. **D**. Original recordings

We also assessed oxidative stress parameters in the mitochondria, as mitochondria are a primary source of reactive oxygen species (ROS) production. All three parameters– GSSG/GSH ratio (Fig. 3I AS−DM: mean ± SEM: 0.002815 ± 0.0003327; AS+DM: 0.003985 ± 0.0004364), H_2_O_2_ levels (Fig. 3J; AS−DM: mean ± SEM: 12.65 ± 1.582 µM; AS+DM: 17.43 ± 1.329 µM), and LPO (Fig. 3K; AS−DM: mean ± SEM: 1.602 ± 0.1505 pg/mg; AS+DM: 2.178 ± 0.1871 pg/mg)—were significantly elevated in the mitochondria of diabetic AS patients compared to those without DM. The increased oxidative status observed in diabetic AS patients was accompanied by an increase in cardiomyocyte F_passive_ beyond the SL of 2.0 µm, which could be normalized to levels comparable to those of the non-diabetic group following in vitro treatment with the antioxidant GSH (Figs. 4A, 4B).

Furthermore, we conducted force measurements in both AS groups using the mitochondria-targeted antioxidant Mito-TEMPO (Figs. 4C, 4D). A decrease in stiffness was also observed in diabetic AS cardiomyocytes following Mito-TEMPO treatment; however, the levels improved but did not quite reach those observed in the treated non-diabetic group (Figs. 4C, 4D).

### Diabetes mellitus impairs the NO-sGC-cGMP-PKG signaling pathway in aortic stenosis patients

**Fig. 5:**
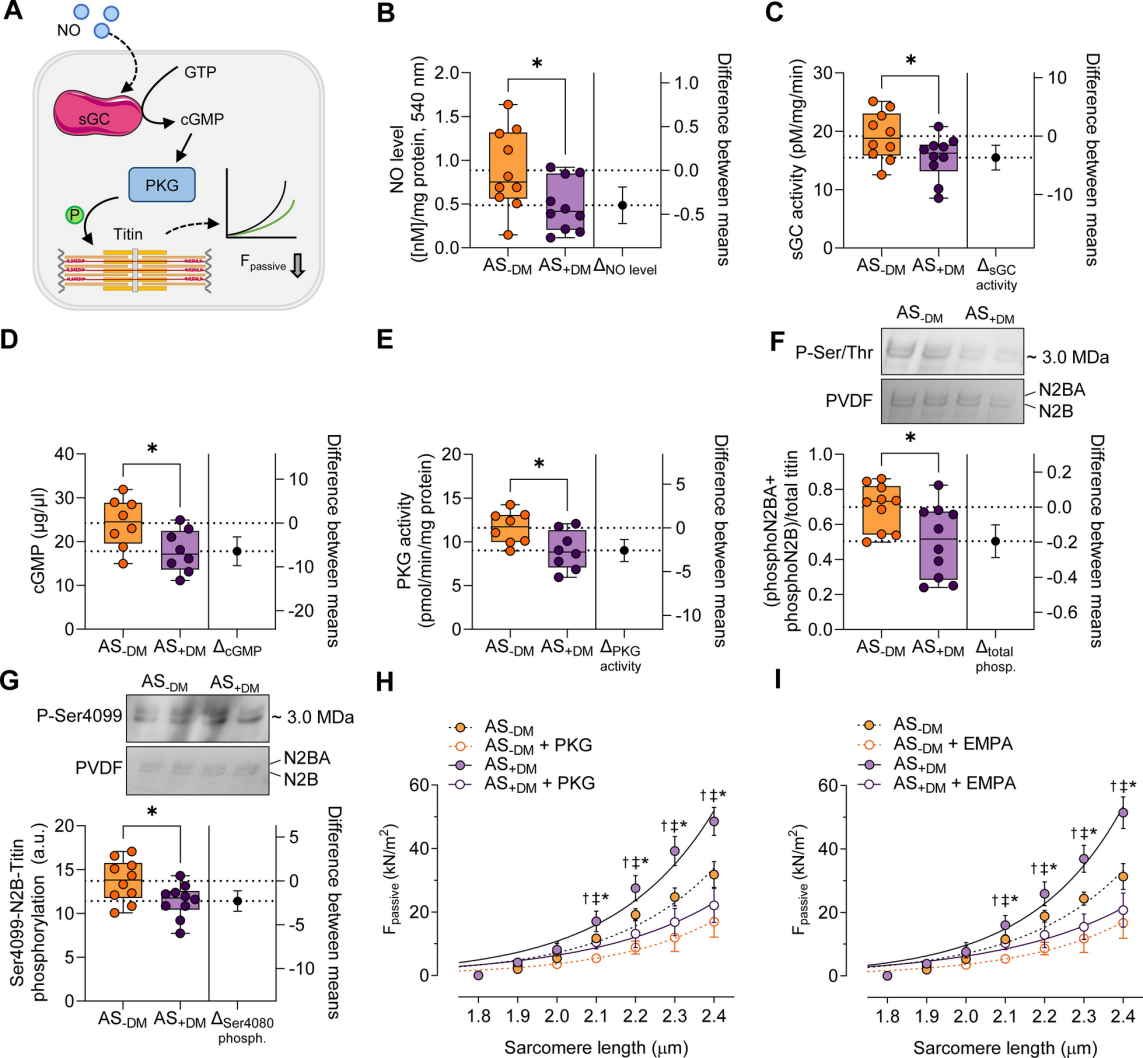
Myocardial NO/sGC/cGMP/PKG signaling pathway in left ventricular biopsies from patients with aortic stenosis (AS), with (+DM) and without concomitant diabetes (−DM). **A** Schematic representation of the myocardial nitric oxide (NO)/soluble guanylyl cyclase (sGC)/cyclic guanosine monophosphate (cGMP)/protein kinase G (PKG) signaling pathway. F_passive_: passive stiffness; GTP: guanosine triphosphate; P: phosphorylation. **B** NO levels. **C** Activity of sGC. **D** Myocardial levels of cGMP. **E.** Activity of PKG. **F.** Ratio of phosphorylated N2B and N2BA titin isoforms to total titin. **G.** Site-specific phosphorylation at Ser4099 of the N2B isoform. Box and whisker plots (indicating median, 25th to 75th percentiles, minimum, and maximum) are utilized on the left axis to represent selected parameters across each group (*n* = 8–10 samples per group). The dashed lines represent the mean values of both groups. *P*-values are from unpaired t-test; *P < 0.05. The right axis shows the difference between the means ± SEM. **H** F_passive_ at SL 1.8–2.4 µm in the presence or absence of PKG. **I** F_passive_ at SL 1.8–2.4 µm in the presence or absence of empagliflozin (EMPA). Curves are second-order polynomial fits to the means (± SEM; *n* = 30–36/5 cardiomyocytes/heart per group). *P < 0.05 AS−DM versus AS+DM, ^‡^*P* < 0.05 AS−DM versus AS−DM after PKG or EMPA treatment, †*P* < 0.05 AS+DM versus AS+DM after PKG or EMPA treatment by one-way ANOVA. *P*-values were corrected for multiple comparisons by the Tukey method

It is likely that elevated oxidative stress negatively affects, directly or indirectly, the bioavailability of nitric oxide (NO), thereby diminishing the PKG signaling pathway (Fig. 5A). In our investigation, we measured NO levels in both AS patient cohorts and observed a significant reduction in NO production among diabetic AS patients compared to their non-diabetic counterparts (Fig. 5B; AS−DM: mean ± SEM: 0.8844 ± 0.1443 nM/mg protein; AS+DM: 0.4868 ± 0.0937 nM/mg protein). This reduction resulted in markedly lower activation of sGC (Fig. 5C; AS−DM: mean ± SEM: 19.17 ± 1.303 pM/mg/min; AS+DM: 15.49 ± 1.172 pM/mg/min), diminished cGMP concentration (Fig. 5D; AS−DM: mean ± SEM: 24.23 ± 2.004 µg/µL; AS+DM: 17.79 ± 1.709 µg/µL), and decreased PKG activity (Fig. 5E; AS−DM: mean ± SEM: 11.59 ± 0.6546 pmol/min/mg protein; AS+DM: 9.014 ± 0.7765 pmol/min/mg protein).

The sarcomeric giant protein titin, which is pivotal for muscle elasticity, plays a crucial role in determining cardiomyocyte F_passive_. Alterations in post-translational modifications, such as phosphorylation, are known to influence myocardial elasticity and, consequently, cardiomyocyte F_passive_. We assessed the total and PKG-dependent phosphorylation status of titin using Western blotting techniques. Our results indicated that total titin phosphorylation was significantly lower in diabetic AS patients compared to non-diabetic AS patients (Fig. 5F; AS−DM: mean ± SEM: 0.6986 ± 0.04135 a.u.; AS+DM: 0.5040 ± 0.06509 a.u). In alignment with this observation, the PKG-dependent phosphorylation status of Ser4099 within the elastic N2-Bus sequence of titin was also reduced in diabetic AS patients relative to non-diabetic AS patients (Fig. 5G; AS−DM: mean ± SEM: 13.71 ± 0.7539 a.u.; AS+DM: 11.42 ± 0.5985 a.u.). This deficit in titin phosphorylation is partially responsible for the increased F_passive_ observed in diabetic AS patients (Fig. 5H).

When PKG was applied in vitro to cardiomyocytes from both AS groups, we noted a reduction in F_passive_ for both cohorts. However, the levels in the treated diabetic AS group remained higher than those in the treated non-diabetic AS patients (Fig. 5H). Recently, we demonstrated that the sodium glucose transporter 2 (SGLT2) inhibitor empagliflozin (EMPA) possesses antioxidative and anti-inflammatory properties, which enhance the NO-sGC-cGMP-PKG signaling cascade [15]. Administration of EMPA to skinned cardiomyocytes from diabetic AS patients resulted in a reduction of elevated F_passive_, though not to the levels observed in non-diabetic AS patients (Fig. 5I).

### Altered PKA and CaMKII in Diabetic AS Patients

**Fig. 6:**
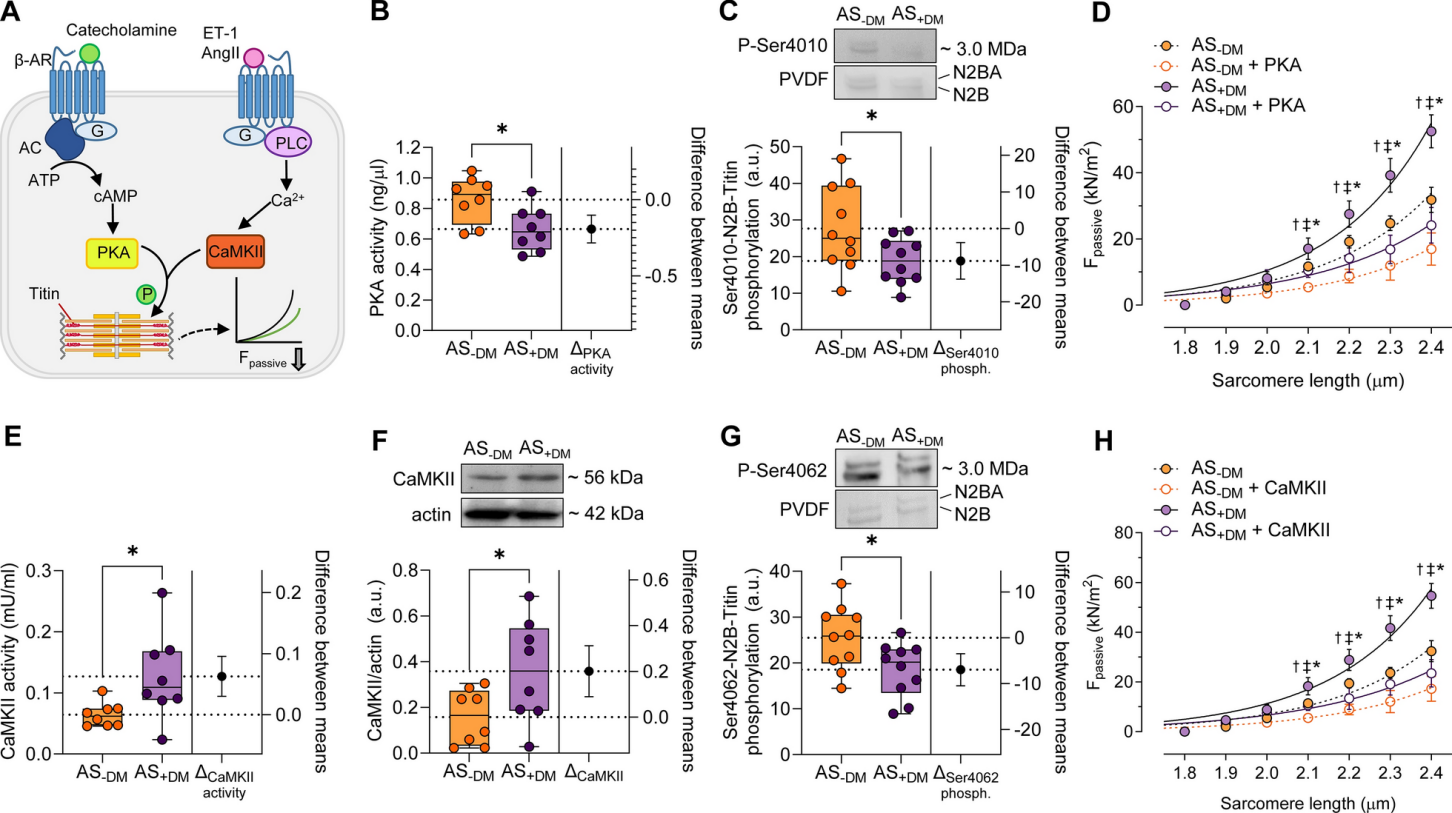
PKA and CaMKII in left ventricular biopsies from patients with aortic stenosis (AS), with (+DM) and without concomitant diabetes (−DM). **A** Schematic representation of the myocardial protein kinase A (PKA) and calcium-calmodulin dependent protein kinase II (CaMKII) signaling pathways. AC: adenylyl cyclase; AngII: angiotensin II; β-AR: β-adrenergic receptor; ATP: adenosin triphosphate; Ca^2+^: calcium; cAMP: cyclic adenosine monophosphate; ET-1: endothelin 1; F_passive_: passive stiffness; G: G protein; P: phosphorylation; PLC: phospholipase C. **B **PKA activity. **C**. Site-specific phosphorylation at Ser4010 of N2B isoform. **D**. F_passive_ at SL 1.8–2.4 µm in the presence or absence of PKA. **E** CaMKII activity. **F** Expression of CaMKII. **G**. Site-specific phosphorylation at Ser4062 of N2B isoform. **H** F_passive_ at SL 1.8–2.4 µm in the presence or absence of CaMKII. Panels B, C, E, F, G Box and whisker plots (median, 25th to 75th percentiles, minimum and maximum) are used on the left axis to represent selected parameters in each groups (*n* = 8–10 samples per group). The dashed lines represent the mean values of both groups. *P*-values are from unpaired t-test; *P < 0.05. The right axis shows the difference between the means ± SEM. Panels D+H. Curves are second-order polynomial fits to the means (± SEM; *n* = 30–36/5 cardiomyocytes/heart per group). *P < 0.05 AS–DM versus AS+DM, ^‡^*P* < 0.05 AS−DM versus AS−DM after PKA or CaMKII treatment, †*P* < 0.05 AS+DM versus AS+DM after PKA or CaMKII treatment by one-way ANOVA. *P*-values were corrected for multiple comparisons by the Tukey method

In addition to PKG, two other protein kinases that phosphorylate titin and thereby influence cardiomyocyte F_passive_ are protein kinase A (PKA) and Ca^2+^/calmodulin-dependent kinase II (CaMKII) (Fig. 6A) [32, 35]. We evaluated their kinase activities, kinase-specific titin phosphorylation, and their effects on F_passive_ in diabetic versus non-diabetic AS patients (Fig. 6). PKA activity (Fig. 6B; AS−DM: mean ± SEM: 0.8585 ± 0.05341 ng/µL; AS+DM: 0.6649 ± 0.05079 ng/µL) was significantly reduced, accompanied by diminished PKA-dependent phosphorylation of Ser4010 within the elastic N2-Bus titin sequence (Fig. 6C; AS−DM: mean ± SEM: 27.65 ± 3.620 a.u.; AS+DM: 18.83 ± 1.965 a.u.) in the diabetic cohort compared to the non-diabetic AS patient group. The increased F_passive_ observed in diabetic patients could be reduced by in vitro PKA treatment, although it could not be normalized to the levels observed in treated non-diabetic AS patients (Fig. 6D). Conversely, CaMKII activity and expression were notably enhanced (Fig. 6E: AS−DM: mean ± SEM: 0.06424 ± 0.006952 mU/mL; AS+DM: 0.1271 ± 0.02542 mU/mL; Fig. 6F: AS−DM: mean ± SEM: 0.1576 ± 0.04235 a.u.; AS+DM: 0.3582 ± 0.07917 a.u.); however, the CaMKII-dependent phosphorylation status of Ser4062 was significantly diminished (Fig. 6G; AS−DM: mean ± SEM: 25.49 ± 2.209 a.u.; AS+DM: 18.52 ± 1.862 a.u.). Similarly, the elevated cardiomyocyte F_passive_ in diabetic patients could be decreased by in vitro CaMKII treatment, but normalization to the levels of treated non-diabetic AS patients was not achieved (Fig. 6H).

## Discussion

In the present study, we investigated the effects of DM on the LV myocardium in patients with AS, with a particular focus on the inflammatory response and oxidative stress, as well as their contributions to cardiomyocyte function, titin phosphorylation, and NO-sGC-cGMP-PKG signaling pathway. Our findings indicate that DM exacerbates inflammation and oxidative stress in AS patients, which is associated with impaired NO-sGC-cGMP-PKG signaling and increased passive tension in cardiomyocytes. Anti-inflammatory and antioxidant therapies may ameliorate these alterations, thereby providing potential therapeutic options for patients with concurrent DM and AS. These conditions are reminiscent of the pathophysiology of HFpEF. With respect to the development of increased passive tension in cardiomyocytes, probably HFpEF and AS share common pathways which are aggravated in the presence of DM.

This interplay between AS and DM may even be more complex. Patients with both DM and AS encounter distinct challenges due to the complex interplay between these conditions. If left untreated, this can lead to significant health complications and an increased risk of mortality [1]. DM not only increases the prevalence of AS but also accelerates its progression and reinforces the underlying pathophysiological processes [4, 36–39]. This accelerated progression is primarily attributed to the heightened inflammatory state and the presence of oxidative stress associated with DM, both of which contribute to the calcification of the aortic valve [40], thereby promoting the development of AS. Furthermore, hyperglycaemia, insulin resistance, dyslipidaemia, and the production and deposition of AGEs trigger inflammatory processes and endothelial dysfunction, which further facilitate the progression of AS [1, 41].

### Inflammatory response in DM and AS

Our current study demonstrates that the LV myocardium of patients with concomitant DM and AS exhibits a pronounced inflammatory response characterized by elevated levels of pro-inflammatory mediators (e.g. HMGB1, calprotectin) and receptors (TLR2, TLR4, RAGE) as well as enhanced NLRP3 inflammasome activation, which is correlated with increased interleukin release. This increased inflammation plays a decisive role in the progression of AS in diabetic patients. The binding of HMGB1 to receptors such as TLR2, TLR4, and RAGE triggers inflammatory pathways that contribute to endothelial dysfunction and vascular inflammation. It has been demonstrated that in diabetic patients, elevated HMGB1 levels are linked to increased inflammation and vascular complications [42, 43]. In diabetic patients, increased expression of TLR2 and TLR4 has been implicated in the pro-inflammatory state [43], which enhances sensitivity to inflammatory mediators and contributes to vascular inflammation. In addition, increased AGE deposition in the myocardium of diabetic AS patients has been reported in several studies [12, 41]. AGEs bind to RAGEs, which interact with various ligands, including HMGB1 and calprotectin, and both were significantly elevated in diabetic AS patients in this study. Elevated RAGE expression in diabetic patients has been associated with the development of endothelial dysfunction [44].

Given that hyperglycaemia in DM promotes the formation and accumulation of AGEs, it activates RAGEs, exacerbating vascular inflammation and leading to a vicious cycle of increased inflammation that contributes to the progression of atherosclerosis and aortic calcification. In our cohort of diabetic AS patients, we observed a notably elevated activation of the NLRP3 inflammasome and increased interleukin release compared to the non-diabetic AS group. Several factors may account for the enhanced activation of the NLRP3 inflammasome by DM. These factors include oxidative stress, as ROS facilitate the assembly of the inflammasome complex [45], and the elevation of inflammatory mediators such as HMGB1 and calprotectin (as noted in our study), which promote interactions with TLRs and RAGE, ultimately triggering NLRP3 expression and activation. Consistent with our findings, Lee et al. have demonstrated that the presence of DM enhances the systemic pro-inflammatory milieu in AS patients by showing increased expression of various markers such as E-selectin, IL-1 receptor, and intercellular adhesion molecule 2, all of which contribute to the escalation of myocardial fibrosis, diastolic dysfunction, and poorer clinical outcomes [46].

### Oxidative stress and cardiomyocyte function in DM and AS

Oxidative stress arises from an imbalance between ROS production and the antioxidant defense system. Our study demonstrates that patients with DM and AS have increased exhibit elevated levels of oxidative stress parameters within the myocardium and mitochondria of the left ventricle. Specifically, we observed an increased ratio of GSSG/GSH in both the myocardium and mitochondria, as well as heightened levels of H_2_O_2_ in these tissues. Additionally, LPO levels were found to be increased exclusively in the mitochondria. It is well-established that oxidative stress and inflammation are intricately linked, both contributing to the deterioration of cardiomyocyte function [22]. Several studies have documented the presence of oxidative stress in the context of DM [47–49]. On the one hand, oxidative stress characterized by elevated levels of superoxide and H_2_O_2_, alongside diminished expression and activity of antioxidant enzymes, has been shown to damage the aortic valve, thereby exacerbating calcification and stenosis [5]. This heightened oxidative environment in diabetic patients may accelerate the progression of AS. On the other hand, ROS-induced damage can impair mitochondrial function, leading to alterations in cardiac metabolism and energy deficits. Mitochondrial dysfunction is a critical factor in diabetic HF, contributing to contractile dysfunction [50]. Furthermore, oxidative stress can modify cardiac proteins directly, impacting their functionality and contributing to impaired contractility. Notably, this includes modifications in proteins involved in Ca^2+^ handling, contractile processes and signaling pathways such as kinases [22, 25]. All these alterations are associated with compromised cardiomyocyte function and increased F_passive_, as multiple studies have indicated that oxidative modifications of titin—such as disulfide bonding and S-glutathionylation of cryptic immunoglobulin domains—correlate with altered F_passive_ properties [24, 51, 52]. Previous work has demonstrated that oxidative modification of kinases implicated in titin phosphorylation is accompanied by increased cardiomyocyte F_passive_ [15, 27, 53]. Moreover, there exists an interplay between ROS and AGEs in the context of DM, as ROS can accelerate the formation of AGEs. These glycated proteins, in turn, perpetuate ROS production, leading to further cellular injury [54].

### NO-sGC-cGMP-PKG signaling pathway in DM and AS

Oxidative stress is implicated in vascular dysfunction and thus exerts a direct influence on the bioavailability of NO, which is essential for the activation of sGC and the subsequent PKG signaling pathway. In DM, hyperglycaemia-induced oxidative stress results in the production of ROS that scavenge NO, leading to a reduction in NO bioavailability. This decrease impairs sGC activation, culminating in diminished cGMP production [55]. The NO-sGC-cGMP-PKG signaling pathway is critical for various cellular processes, including vasodilation, modulation of cardiac contractility and relaxation, alteration of cardiomyocyte F_passive_, as well as the suppression of hypertrophy and pathological remodeling [56, 57]. Notably, reduced PKG activity associated with decreased cGMP levels has been observed across a spectrum of heart diseases in both humans and animal models, including HF with preserved ejection fraction (HFpEF), hypertrophic cardiomyopathy, myocardial infarction-induced HF with reduced LV ejection fraction (HFrEF), and AS [13–19]. In the settings of DM and AS, the NO-sGC-cGMP-PKG pathway is significantly compromised, contributing to the pathophysiology observed in these conditions, including reduced titin phosphorylation and increased F_passive_. A prior study indicated that a cohort of HFpEF patients, characterized by a high prevalence of obesity and DM, exhibited heightened oxidative stress, as evidenced by elevated myocardial nitrotyrosine expression. This was associated with lower cGMP concentrations and reduced PKG activity, thereby resulting in increased cardiomyocyte F_passive_ when compared to patients with AS [13]. In the present study, NO production (as reflected by total nitrite/nitrate content) and the myocardial 3-nitrotyrosine levels were not further elevated by DM, instead we found reduced levels as compared to non-diabetic AS patients. Recent work from other groups suggests that excessive nitrosative stress and accumulation of S-nitrosylated proteins play a causative role in the pathogenesis of HFpEF [58]. Our data does not confirm nitrosative stress, in addition to oxidative stress, as a confounding pathomechanism of DM in the exacerbation of myocardial stiffness. However, our results underscores that the presence of comorbidities such as obesity and DM correlates with a more pronounced impairment of the NO-sGC-cGMP-PKG signaling pathway. The decreased bioavailability of NO, and consequently reduced sGC activity, cGMP levels, and PKG activity, are responsible for the phosphorylation deficit of titin observed in a majority of heart diseases [15–20]. In diabetic patients, diminished sGC expression has been linked to reduced PKG-dependent titin phosphorylation at Ser4099 within the N2B-us region increasing cardiomyocyte F_passive_ [11]. The reduction of PKG-dependent phosphorylation at the N2B-us region may significantly contribute to the augmented diastolic stiffness observed in failing hearts, as ex vivo incubation with PKG has been shown to rectify the increased F_passive_ of skinned cardiomyocytes in human cases, including those with HFpEF and AS, with or without DM [12, 13, 59–61], as well as in animal models of HFpEF, diastolic dysfunction, and cardiac hypertrophy [14, 16, 18, 20, 53]. Furthermore, previous studies have demonstrated that the oxidation of PKG also contributes to the phosphorylation deficit of titin, subsequently raising cardiomyocyte F_passive_ [15, 16, 27, 53].

### Titin and myocardial stiffness

The giant sarcomeric protein titin plays a pivotal role in modulating diastolic function by regulating myocardial stiffness and elasticity. Alterations in titin isoform expression and post-translational modifications, particularly phosphorylation, have been implicated in both DM and AS, especially in the development of diastolic dysfunction and HFpEF. Titin functions as a molecular spring that determines cardiomyocyte F_passive_ and, consequently, influences cardiac mechanical properties. Within the heart, there are two predominant isoforms: the longer N2BA isoform, which is compliant and more elastic, and the shorter N2B isoform, which is stiffer and thus less elastic [59]. Previous research has yielded varying results regarding titin isoform shifting in AS: the N2BA:N2B expression ratio was found to be higher in AS patients than in control hearts, [61], unaltered in diabetic AS compared to non-diabetic AS patients [12], or reduced in AS patients relative to control donor samples [62]. One study demonstrated an increased proportion of the compliant N2BA isoform in right atrial auricles from diabetic patients, along with increased cardiomyocyte F_passive_ compared to controls [11]. The modulation of F_passive_ through post-translational modifications of titin alters its mechanical properties in response to physiological and pathophysiological stimuli [59]. Phosphorylation of titin's spring elements, particularly within the cardiac-specific N2B unique sequence (N2B-us) and the region rich in proline, glutamine, valine, and lysine (PEVK), by kinases such as CaMKIIδ, PKA, PKG, and protein kinase Cα (PKCα) can decrease titin-based passive tension and consequently reduce cardiomyocyte F_passive_ (in the case of PKA, PKG, and CaMKIIδ) or increase titin-based passive tension and thereby elevate cardiomyocyte F_passive_ (in the case of PKCα) [32, 35, 63–65]. Our findings of overall reduced titin phosphorylation, along with diminished PKG- and PKA-dependent titin phosphorylation at Ser4010 and Ser4099 within the N2B-us region in diabetic compared to non-diabetic AS patients, are partially associated with increased cardiomyocyte F_passive_. In vitro treatment of cardiomyocytes with PKG and PKA respectively reduced F_passive_ in both AS groups, although the levels in treated diabetic AS patients remained elevated compared to those in non-diabetic AS patients, supporting the notion that diabetic AS patients exhibit more pronounced changes due to heightened oxidative stress and inflammatory responses. Hopf et al. demonstrated in right atrial samples from diabetic patients a phosphorylation deficit at Ser4099 due to decreased PKG activity and hyperphosphorylation at Ser11878 in the PEVK segment attributable to increased PKCα activity, accompanied by increased F_passive_ compared to the non-diabetic control group [11]. Furthermore, fibrosis and interstitial collagen deposition can exacerbate diastolic function as they contribute to myocardial diastolic F_passive_ in conjunction with titin-derived cardiomyocyte F_passive_. It is well established that patients with DM exhibit excessive fibrosis and higher deposition of AGEs [34], and this phenomenon is even more pronounced in diabetic AS compared to non-diabetic patients. Together with the hypophosphorylation of titin, these factors contribute to the worsening of diastolic LV dysfunction [12]. Moreover, to further characterize the extent and distribution of myocardial fibrosis, future studies should incorporate dedicated histological staining techniques such as Masson’s trichrome or Sirius Red.

### Therapeutic implications and clinical relevance

The interplay between DM and AS presents unique clinical challenges, as the coexistence of these conditions complicates treatment strategies due to their reciprocal exacerbation of progression. Understanding the interconnected pathophysiological mechanisms—characterized by increased inflammation and oxidative stress, reduced titin phosphorylation, and elevated cardiomyocyte F_passive_—is crucial for developing effective treatment strategies for affected patients. Our findings indicate that the heightened inflammation and oxidative stress in AS patients with DM are associated with a more pronounced increase in cardiomyocyte F_passive_ compared to their non-diabetic counterparts. Ex vivo anti-inflammatory and antioxidant treatment of cardiomyocytes significantly normalized F_passive_ to levels comparable to those observed in the non-diabetic group. Targeting these pathways may confer therapeutic benefits aimed at slowing the progression of AS in patients with DM. Additionally, treatment of skinned cardiomyocytes with EMPA resulted in a reduction of the elevated F_passive_ in both patient groups. Recent studies have demonstrated that EMPA mitigates oxidative stress and inflammation, enhances NO-sGC-cGMP-PKG signaling cascade, improves titin phosphorylation, and reduces cardiomyocyte F_passive_ in patients with HFpEF [15]. In addition, EMPA have shown to improve diastolic stiffness and diastolic function in human failing hearts and murine animal models which has pleiotropic effects on the myocardium due to improved phosphorylation of myofilament regulatory proteins [66]. Moreover, Matsuda et al. have shown that the SGLT2 inhibitor (SGLT2i) dapagliflozin mitigated oxidative stress and reduced inflammatory response in a rabbit model of cardiac surgery-associated acute kidney injury [67]. By reducing inflammation, oxidative stress, and myocardial fibrosis, SGLT2i such as EMPA and similar agents may alleviate the adverse effects of DM on cardiac function in AS patients. However, identifying suitable candidates for SGLT2i therapy in the context of AS is crucial. Clinical studies to date have focused primarily on patients with HF, particularly those with reduced ejection fraction (HFrEF; EMPEROR-Reduced trial [68]), HFpEF (EMPEROR-Preserved trial [69]) and DM patients (EMPA-REG OUTCOME [70]), and have demonstrated cardiovascular benefits in these patient groups. Recently, Shah et al. have shown in a retrospective, observational study that SGLT2i may slow the progression of non-severe AS [71]. However, the efficacy and safety of SGLT2i in AS patients with and without DM has not yet been sufficiently investigated. In addition, the heterogeneity of AS—ranging from mild to severe—requires a differentiated approach when initiating therapy. Patients with advanced AS may have altered hemodynamics, making the hemodynamic effects of SGLT2i, such as diuresis and blood pressure reduction, potentially detrimental. Therefore, careful assessment of AS severity, comorbidities, especially DM, and overall cardiovascular status is essential before considering SGLT2i therapy for these patients cohort. Moreover, recent data have shown that optimized in-hospital guideline-directed medical therapy (GDMT) scores, including SGLT2i, are associated with improved outcomes in HF patients, even after readmissions, highlighting the importance of personalized and timely pharmacological interventions [72, 73]. In this context, SGLT2i have emerged as a cornerstone therapy across the HF spectrum, including HFpEF, with increasing endorsement in major international guidelines [74].

In terms of treatment, aortic valve repair, either surgical replacement (AVR) or catheter-based aortic valve implantation (TAVI), remains the primary intervention for patients with severe AS; however, Lindman et al. have demonstrated that DM is linked to increased morbidity and mortality following surgical AVR (SAVR) [75]. With regard to pharmacological interventions, there is currently no established medical therapy that can halt or reverse the progression of AS. Nonetheless, ongoing clinical trials are exploring potential treatments. For example, the EVOID-AS trial is investigating the efficacy of evogliptin in reducing the progression of aortic valve calcification in patients with mild to moderate AS [76]. Evogliptin is a dipeptidyl peptidase-4 (DPP-4) inhibitor utilized in the treatment of DM [77]. Recent research involving ZSF1 rats, a model of metabolic syndrome and ventricular stiffness, has demonstrated that the DPP-4 inhibitor linagliptin reduces LV stiffness by decreasing cardiac fibrosis and cardiomyocyte F_passive_. This reduction is attributed to titin isoform switching from the stiffer N2B to the more compliant N2BA, as well as increased phosphorylation of total titin and specifically its N2B-us region (Ser4080 and Ser3391) [78]. Another DPP-4 inhibitor, sitagliptin, has been shown to decrease LV cardiomyocyte F_passive_ and enhance global LV performance due to elevated cGMP levels, PKG activity, and improved titin phosphorylation in a mouse model of obesity and DM [79]. Collectively, targeting the pathways of oxidative stress and inflammatory responses in patients with concomitant AS and DM may present novel therapeutic options for slowing AS progression and alleviating HF symptoms by enhancing titin phosphorylation and mitigating cardiomyocyte F_passive_. Furthermore, monitoring biomarkers of oxidative stress and inflammation may assist in risk stratification.

## Conclusions

This study demonstrates that DM exacerbates inflammation and oxidative stress in patients with AS, resulting in impaired cardiac function and increased F_passive_ of cardiomyocytes. This process is reminiscent of the pathophysiology of HFpEF. Elevated levels of pro-inflammatory mediators and diminished NO bioavailability compromise the NO-sGC-cGMP-PKG signaling pathway, which is critical for the health of cardiomyocytes. Our findings suggest that targeting inflammation and oxidative stress through anti-inflammatory and antioxidant therapies, in addition to the use of SGLT2-inhibitors such as empagliflozin, may provide potential therapeutic strategies for managing patients with concurrent DM and AS. Further research is necessary to refine these approaches and enhance outcomes in this high-risk population.

## Data Availability

All data generated or analysed during this study are included in this published article.

## Abbreviations

AGE: Advanced glycation end products
AS: Aortic stenosis
ATPG: Aortic transvalvular pressure gradient
AVAi: Aortic valve area index
AVR: Aortic valve replacement
CaMKII: Calcium-calmodulin dependent kinase II
cGMP: Cyclic guanosine monophosphate
DM: Diabetes mellitus
DPP-4: Dipeptidyl peptidase-4
ELISA: Enzyme-linked immunosorbent assay
EMPA: Empagliflozin
F_passive_: Passive stiffness
GSH: Reduced glutathione
GSSG: Oxidized glutathione
HF: Heart failure
HFpEF: Heart failure with preserved ejection fraction
HFrEF: Heart failure with reduced ejection fraction
HMGB1: High mobility group box protein 1
HRP: Horse radish peroxidase
H_2_O_2_: Hydrogen peroxide
IL-1/-6/-18: Interleukin-1/-6/-18
LPO: Lipid peroxidation
LV: Left ventricular
LVMi: Left ventricular mass index
N2B-us: N2B unique sequence
NF-κB: Nuclear factor kappa B
NLRP3: NOD-like receptor protein 3
NO: Nitric oxide
PEVK: Region rich in proline, glutamine, valine, and lysine
PKA: Protein kinase A
PKG: Protein kinase G
RAGE: Receptor for advanced glycation end products
ROS: Reactive oxygen species
SEM: Standard error of the mean
sGC: Soluble guanylyl cyclase
SGLT2: Sodium glucose transporter 2
SGLT2i: Sodium glucose transporter 2 inhibitor
SL: Sarcomere length
TLR2/4: Toll-like receptor 2/4
